# Cognitive success: instrumental justifications of normative systems of reasoning

**DOI:** 10.3389/fpsyg.2014.00625

**Published:** 2014-07-01

**Authors:** Gerhard Schurz

**Affiliations:** Department of Philosophy, Heinrich-Heine University of DuesseldorfDuesseldorf, Germany

**Keywords:** is and ought, normative accounts of rationality, means-end inference, cognitive success, general vs. locally adaptive rationality

## Abstract

In the first part of the paper (sec. 1–4), I argue that Elqayam and Evan's (2011) distinction between normative and instrumental conceptions of cognitive rationality corresponds to deontological vs. teleological accounts in meta-ethics. I suggest that Elqayam and Evans' distinction be replaced by the distinction between a-priori intuition-based vs. a-posteriori success-based accounts of cognitive rationality. The value of cognitive success lies in its instrumental rationality for almost-all practical purposes. In the second part (sec. 5–7), I point out that the Elqayam and Evans's distinction between normative and instrumental rationality is coupled with a second distinction: between logically general vs. locally adaptive accounts of rationality. I argue that these are two *independent* distinctions that should be treated as independent dimensions. I also demonstrate that logically general systems of reasoning can be instrumentally justified. However, such systems can only be cognitively successful if they are paired with successful *inductive* reasoning, which is the area where the program of adaptive (ecological) rationality emerged, because there are no generally optimal inductive reasoning methods. I argue that the practical necessity of reasoning under *changing* environments constitutes a dilemma for ecological rationality, which I attempt to solve within a *dual* account of rationality.

## Introduction: recent criticisms of normative systems of reasoning in psychology

According to a common conception (Elqayam and Evans, 2011, p. 234), classical logic was the dominant normative standard of rational thinking in cognitive psychology until the 1960s. When psychologists discovered empirically that, in many domains, human reasoning did not accord with the principles of logic (e.g., Wason, 1966), these findings were interpreted as signs of human irrationality (cf. Evans, 2002). Beginning in the 1970s this interpretation came increasingly under attack by authors who demonstrated that deviations from classical logic can nevertheless be rational (e.g., Cohen, 1981; Gigerenzer, 1991; Oaksford and Chater, 1994). For example, when conditionals are uncertain, the optimal rules of conditional reasoning are no longer classical (see section Instrumental Justification of Deductive Reasoning). Some authors suggested that psychologists should adopt a different normative system, as an alternative to classical logic, such as, for example, Bayesian probability theory or decision theory (e.g., Oaksford and Chater, 1991, 2007). However, human reasoning has been observed to deviate from the norms of probability and decision theory, too (Kahneman and Tversky, 1972; Barbey and Sloman, 2007). Therefore other authors suggested that certain forms of “adaptive” or “instrumental” rationality do not presuppose any normative system at all; rather they can and should be studied in a purely descriptive way (e.g., Evans and Over, 1996; Gigerenzer et al., 1999). A clear exposition of this position is given in Elqayam and Evans (2011). I take this position as a starting point for my critical discussion of the notions of rationality that underlie the psychological debate on norms of reasoning.

According to Elqayam and Evans (2011, p. 234), *prescriptive normativism* is the view that human thinking *should* be evaluated against (the rules of) a normative system, *S*, and *ought* to conform to it, where S is a general system of reasoning such as logic, probability theory, or decision theory^1^. Elqayam and Evans launch three major criticisms against prescriptive normativism: (1) First, there are different mutually competing normative systems of reasoning, such as classical vs. non-classical logics, frequentistic vs. Bayesian probability theory, probability theory vs. fuzzy logic, etc. This leads to the problem of “arbitration,” i.e., of deciding between different normative systems. For Elqayam and Evans it is more-or-less impossible to give an objective or unbiased approach to this problem, because normative systems understand their “norms” of reasoning as *fundamental* norms, being based on more-or-less *a-priori* intuitions which are not capable of further *rational* justification^2^. (2) Second, the endeavors of many psychologists to select one of these normative systems on *empirical* grounds are typically based on *is-ought fallacies*. According to a famous philosophical doctrine that goes back to Hume (1739/40, part 1, §1), it is logically impossible to infer an Ought from an Is. (3) Elqayam and Evans recognize instances of *ought-is fallacies* in psychological research, in which psychologists infer incorrectly from their preference for a certain normative system S that a certain theoretical interpretation of people's empirical cognitive behavior is “correct,” due to its coherence with the rules of reasoning prescribed by system S—a position which Elqayam and Evans call “empirical normativism” (Elqayam and Evans, 2011, p. 244, 234). In this way normative prescriptivism introduces *biases* which hinder empirical research.

Because of these problems Elqayam and Evans argue that psychologists of reasoning would be better off if they gave up normative prescriptivism and dispensed with appeals to any normative system whatsoever. They call this opposite position *descriptivism* and mention Gigerenzer and Todd's conception of *adaptive* (ecological) *rationality* as well as Evans and Over's *instrumental rationality* as prototypes of this position (Elqayam and Evans, 2011, P. 246f)^3^. For Evans and Over, a method of reasoning or decision-making is instrumentally rational if it is reliable and efficient *for* achieving one's *subjective goals* (1996, p. 8). Adaptive rationality is considered a kind of instrumental rationality which emphasizes the dependence of the optimal means of achieving one's goals on the given environment; so cognition can only be instrumentally rational if it is ecologically adapted. Like Evans and Over (1996), Todd and Gigerenzer (2012, p. 15) criticize the purported “a-priori” nature of normative systems and argue that the fitness of cognitive methods should be empirically tested in natural environments. Elqayam and Evans (2011, p. 247f) assure readers that their descriptivist position does not exclude normative recommendations entirely from the field of cognitive psychology. However, they argue that all that can be generally said about “rational thinking” (thereby quoting Baron, 2008) is that rational thinking is “whatever kind of thinking helps people to achieve their goals.” This sounds very close to the pragmatist philosophy of William James.

In the following sections, I will try to embed the non-normativist positions of Evans and Over (1996), Elqayam and Evans (2011), and Todd and Gigerenzer (2012) into a more general philosophical framework. I will suggest replacing the conception of “normativist” vs. “instrumentalist” rationality by two independent distinctions: the distinction between intuition-based vs. success-based conceptions of rationality, and the distinction between logico-general vs. local-adaptive conceptions of rationality.

## Deontological vs. consequentialist justifications of norms in the context of the is-ought problem

The distinction between normative vs. instrumental rationality is related to a standard distinction in meta-ethics: that between deontological vs. consequentialist justifications of norms. In *deontological* systems of ethics, the normative basis of justification consists of certain *fundamental norms*, which assert that certain general forms of action are *categorically* (i.e., unconditionally) obligatory or ethically *good in themselves*. In contrast, in *consequentialist* systems of ethics, actions are justified as normatively right because of the *value* of their consequences, or at least of those consequences that were foreseeable by the actor (Broad, 1930; Anscombe, 1958; Birnbacher, 2003; ch. 4). Consequentialist ethics are further divided into two groups: in *value-consequentialist* (or non-teleological) ethics, actions are normatively right because their consequences are ethically valuable, while in *teleological* ethics, actions are normatively right because their consequences promote the satisfaction of extra-ethical values, which consist in the *factual* goals of people, ultimately the avoidance of pain and achievement of pleasure (cf. Frankena, 1963, ch. 2). The most famous historical example of a deontological position is Kant's *categorial imperative*, which requires one to treat all morally relevant subjects equally and seems to be ethically right quite independently from its consequences. The most famous historical example of a teleological position is Benthem's and Mill's *utilitarianism*, for which an action is ethically right just in case it results in the “greatest happiness of the greatest number.” While utilitarianism is an *altruistic* principle, its egoistic variant is *egoistic hedonism*, according to which an action is right for an agent if it maximizes the agent's *own* personal pleasure.

Let us discuss these ethical positions in the light of Hume's thesis that norms and ethical values cannot be logically derived from descriptive facts. Contemporary attempts to prove Hume's thesis by means of modern logic have faced surprising difficulties. These difficulties derive from the paradox of Prior (1960), which is based on two facts:

- From *purely descriptive* premises, for example ¬p (e.g., “I am not poor”) one may derive *mixed* statements such as ¬p∨Pq, with “P” for “is permitted” (e.g., “I am not poor or stealing is permitted”).
- From the mixed statement ¬p∨Pq together with the descriptive premise p one can derive the *purely normative* statement “Pq.”

Prior argued that if mixed statements count as descriptive, then (ii) counts as an is-ought inference, and if mixed statements count as normative, then (i) counts as an is-ought inference. So it seems that is-ought inferences result in either case (which constitutes Prior's paradox). The major insight that emerged from this paradox was that an adequate explication of Hume's is-ought thesis must be based on the *threefold* division of statements into purely descriptive, mixed, and purely normative. Based on this insight, Schurz (1997) proved that the following two versions of Hume's thesis hold in all standard logical systems of multi-modal first-order logic:

- (H1) No non-logically true purely normative conclusion can be derived from a consistent set of purely descriptive premises.
- (H2) Every mixed conclusion which follows logically from a set of purely descriptive premises is normatively irrelevant in the sense that all of its normative subformulas are replaceable by other arbitrary subformulas, salva validitate of the inference.

Thesis (H1) entails the *inverse* Hume thesis (H3) which says that no non-tautologous descriptive statement can be logically inferred from a consistent set of purely normative premises^4^.

In the light of the logical is-ought gap described by Hume, the explained positions of deontological, value-consequentialist, and teleological ethics describe the three major ways in which normative systems can be justified. Norms cannot be derived from facts alone, but they can be justified by deriving them from either (i) other norms having the status of *fundamental* norms (as in deontological ethics), (ii) fundamental ethical values, as in value-consequentialist accounts, or (iii) fundamental extra-moral values which are given by human goals or interests, as in teleological accounts. All three models of justification are based on a so-called *means-end* inference, which has the following form:

(1) *Means-end inference*:
  Normative premise: A is a (fundamental) norm or value.
  Descriptive premise: B is a necessary (or optimal) means for achieving A.
  Normative conclusion: B is a derived norm or value.

This form of means-end inference is accepted as *analytically* valid within more-or-less all kinds of ethical theories, whether they are deontological, value-consequential, or teleological. Here “analytically valid” means “conceptually valid,” i.e., “valid because of the meaning of the involved terms,” but not “logically valid,” i.e., “valid solely because of the meaning of the involved logical terms” (cf. Schurz, 2013, ch. 3.3–3.4). For example, “This is round, therefore it has no edges” is an analytically but not logically valid argument. Moreover, the analytical validity of the means-end inference holds only for necessary and optimal means to an end, but fails for sufficient means^5^.

The second premise of the means-end inference, concerning the means-end relation, is a *factual* statement, expressing the results of empirical research. Thus, the means-end principle explains how the findings of empirical scientists can become practically relevant *without* committing an is-ought fallacy: empirical findings allow one to derive a multitude of derived norms from a small set of fundamental norms or values. The latter ones cannot be established by empirical science (following from Hume's is-ought thesis), but are given to the scientist by *extra-scientific* institutions, e.g., by politicians or by the society as a whole (Schurz, 2010, §6).

With help of means-end inferences one can only prove *hypothetical* (conditional) norms or values, i.e., *implications* of the form “if X is accepted as a norm or value, then Y is also normatively required or valuable”; but one can never justify *categorial* (unconditional) norms in this way (cf. Schurz, 1997, theorem 6, p. 132). For the latter purpose one needs additional premises. They come either in the form of *fundamental norms* or values, or, as in deontological or value-consequentialist accounts, in the form of factual interests of people together with *fundamental* ethical *is-ought* or is-value *bridge principles*, as in teleological theories. The fundamental bridge principle of hedonistic or utilitarian ethics, for example, states that “if the realization of a state of affairs p serves the interests of (some, most, or all) human beings, then p is valuable and ought to be realized.” In deontological and value-consequentialist ethics, the most fundamental norms and values are assumed to be justified by *a-priori* intuition. However, teleological theories also contain such an element of a-priori intuition, in the form of a presupposed is-ought (or is-value) bridge principle. Bridge principles of this sort cannot be justified by logical inference (Hume's insight), nor by arguing that they are “valid by definition” (Moore's insight)^6^; they are often controversial and are accepted only in some but not in all ethical theories.

## A-priori intuition-based vs. a-posteriori success-based accounts of rationality

I will now try to connect the psychological distinction between normative and instrumental rationality to the philosophical framework of deontological, value-consequential, and teleological accounts in ethics, and evaluate the former distinction in the light of the latter. Obviously the position of Elqayam and Evans (2011) is a kind of teleological one, but exactly which one is not entirely clear, at least not for me. Nor is it prima facie clear which position is exactly criticized in their arguments against normative rationality—all non-teleological positions, or only certain ones? Let's see.

Elqayam and Evans understand the rules of a normative system of reasoning S as “evaluative” norms. They assume that evaluative norms are based on a-priori intuition, being unamenable to further justification. From a philosophical viewpoint, this view of (evaluative) norms is too narrow, since normative systems (be they deontological or value-consequentialist) *do* contain a multitude of *derived* norms, which are justified as (optimal or necessary) means to satisfying certain fundamental norms. For example, for Elqayam and Evans “poverty should not exist” is an evaluative norm (Elqayam and Evans, 2011, p. 236), but this norm is instrumental for the more fundamental norm that people should not suffer. Just the same is true for normative systems of reasoning: the fact that a general system of reasoning S such as logic or probability theory is accepted as a normative standard does not imply that reasoning in accord with S can only be justified by “a-priori intuition.” Different ways of justifying the rules of logic or probability theory in terms of more fundamental norms, such as cognitive success or truth-conduciveness will be discussed in the section on General vs. Locally Adapted Rationality.

There is, however, a philosophical position to which Elqayam and Evan's criticism does indeed apply. A well-known example of this position is Cohen's account of rationality (1981). For Cohen, rules of logical reasoning such as Modus Ponens or Modus Tollens are based on a-priori intuitions about correct reasoning. If human reasoning deviates from the rules of logic, this could mean for Cohen that these people have different a-priori intuitions about correct reasoning. So they are not irrational, but their reasoning is merely based on a different norm of rationality. Cohen understands his position as a generalization of Goodman's and Rawls' coherentistic conception of a “reflective equilibrium,” which involves the balancing of general intuitions about correct rules and particular intuitions about rule-instances (Goodman, 1955; Rawls, 1971). I propose to call this family of positions “a-priori intuition-based” conceptions of rationality, which I set in opposition to *a-posteriori success-based* conceptions of rationality.

Intuition-based conceptions base rationality on a motley “stew” of intuitions, including intuitions about the correctness of cognitive rules such as Modus Ponens in logic or Bayes' theorem in probability theory. These intuitions are “subjectively a-priori” in the sense that they are taken as primitively given, incapable of further justification, although they can vary between different subjects. For example, religious people would consider different rules of reasoning as “intuitively rational,” compared to non-religious people. It is therefore unavoidable that this conception of rationality must lead to a strong form of *cognitive relativism*, which has in particular been worked out by Stich (1990).

The notion of “prescriptive normativism” as characterized by Elqayam and Evans (2011) or Evans and Over (1996, p. 8) seems to correspond to a-priori intuition-based conceptions rationality. I agree with Elqayam and Evans' criticism of these positions: they take unreliable subjective intuitions as sacrosanct and thereby hinder rational criticism and scientific progress. However, the opposite of a-priori intuition-based conceptions of rationality are not “descriptive” conceptions of rationality (whatever these may be), but rather a-posteriori success-based conceptions of rationality, which evaluate systems of reasoning in terms of the cognitive *value* of their *consequences* in the given *environment*.

The emphasis of the *local adaptivity* of successful reasoning systems, i.e., the dependence of their value on the environment in which they are applied, is a central insight of the research program of ecological rationality. Todd and Gigerenzer (2012, p. 15) write: “We use the term *logical rationality* for theories that evaluate behavior against the laws of logic or probability rather than success in the world,” while “The study of ecological rationality is about finding out which pairs of mental and environment structures go together.” Todd and Gigerenzer's understanding of “logical rationality” matches our notion of a-priori intuition-based accounts of rationality, and their notion of ecological rationality fits with our understanding of a-posteriori success-based accounts, except that I support a dualist standpoint (similar to Evans, 2003), according to which not *only* locally adapted but also certain general reasoning methods can be justified in this success-based way (see the section on General vs. Locally Adapted Rationality).

In the light of contemporary epistemology (cf. Greco and Turri, 2013), a-priori intuition-based accounts are *internalist* accounts of rationality, because they understand the rationality of a cognitive act as an internal property of the underlying cognitive process of the agent, independent from the environment. What these accounts have in common with deontological ethics is that they evaluate the moral rightness of an act solely based on the properties and intentions of the actor at the time of acting, independent from its consequences. In contrast, a-posteriori success-based accounts are *externalist* accounts of rationality, inasmuch as the success of a cognitive act depends on its consequences in the given environment; this is what these rationality accounts have in common with *consequentialist* ethics.

I do not deny that a-posteriori success-based accounts of rationality also involve *some* elements of intuition. But their intuitive elements can be narrowed down to a few fundamental intuitions about human *goals* whose realization are assumed to be *valuable* (which is a fact-value bridge principle of the explained sort). What a-posteriori accounts reject is reliance on *epistemic correctness intuitions*, i.e., intuitions about the epistemic correctness or plausibility of rules of reasoning. In a-posteriori accounts, all epistemic correctness claims of this sort have to be justified by means-end inferences, which attempt to show that the respective rules are instrumental for attaining the assumed goals in the assumed class of environments.

The notion of *success* contains an objective component (success *in* the given environment) as well as a subjective component (success *for* assumed goals). As long as the “goals” *for* an action are not specified, it is prima facie *unclear* what is meant by “success.” Todd and Gigerenzer avoid making any general statement about what the success of cognitive methods consists in. In all of their experiments, however, they assume that the success of a cognitive method increases with the frequency of its “correct” or empirically true inferences (or predictions), and decreases with the cognitive costs of the method, in terms of necessary information search and computation time. This understanding of “cognitive success” is widely accepted in cognitive science. Philosophers often neglect the dimension of cognitive costs and define *truth-conduciveness* (attainment of true and avoidance of false beliefs) as the fundamental epistemic goal (David, 2005). A prominent variant of this position is *reliabilism* (cf. Goldman, 1986; Schurz and Werning, 2009)^7^.

## Truth-conduciveness and cognitive success: instrumentally rational for almost all purposes

I suggest that the fundamental *goal* of cognitive methods in a-posteriori accounts of rationality should be characterized as the maximization of cognitive success, in the explained sense of finding many possibly relevant truths with little cognitive effort. While this position would find many friends within contemporary epistemology, the notion of “truth” seems to be less popular in cognitive psychology. In their reply to Schurz (2011a), Elqayam and Evans (2011, p. 278f) reject truth as the general goal of reasoning, in favor of an unspecific notion of “instrumental rationality,” which is relativized to arbitrary goals. Before we discuss this position, let us analyze the goal of truth-conduciveness or cognitive success in the light of the preceding discussion of positions in ethics. Two general views are possible: (1) One may understand truth-conduciveness as a fundamental epistemic value, incapable of further justification; this understanding corresponds to a value-consequentialist position. (2) One may deny that truth-conduciveness is an “intrinsic value,” but understand the value of cognitive success instrumentally, in terms of its usefulness for the achievement of some given extra-epistemic (or practical) purposes, whatever these purposes may be. The latter viewpoint corresponds to the *teleological* position of *instrumental* rationality, which underlies the views of Elqayam and Evans, Gigerenzer and Todd, and perhaps the majority of psychologists.

First of all, I wish to point out that although instrumental norms or values are *hypothetical* in the sense explained in sec. 2, they are not “descriptive,” but nevertheless possess normative or evaluative content. Recall that, although the second premise of the means-end inference (1) is descriptive, its conclusion is normative or evaluative: it inherits this status from the first premise which asserts that something is a fundamental norm, value, or goal. This is also true when the fundamental value is given by the factual subjective goal of one or many persons (together with a fact-value bridge principle^8^). For example, if it is a fundamental value *for me* to protect the environment, then it is a derived value for me to support Greenpeace.

Secondly, Elqayam and Evans' conception of instrumental rationality may be relativized to *any purpose whatsoever*. They endorse the view that “rational thinking is whatever kind of thinking best helps people achieve their goals” (Elqayam and Evans, 2011, p. 248). Let us ask: doesn't this position imply that rationality, itself, is entirely relative? On closer inspection, the notion of instrumental rationality is semantically ambiguous. At least three different conceptions of instrumental rationality exist in the literature: instrumental rationality as (i) technocratic rationality, (ii) goal-relative rationality, or (iii) general all-purpose rationality. While (i) maintains that instrumental rationality is ideologically biased (Habermas, 1966), (ii) maintains that it is entirely relative: there are as many kinds of instrumental rationality as there are different kinds of human goals (Stich, 1990). Only position (iii)—which I attribute to Evans and Over (1996, p. 8)—maintains that instrumental rationality is general and non-relative.

Is it an unavoidable consequence of the notion of instrumental rationality that it is goal-relative? Do we have for each kind of goal a separate account of rationality? Do environmentalists, warriors, and taxi drivers, etc. each employ different methods of rational reasoning? *This seems to be entirely wrong*. In this section, I present a simple argument that shows that there is a form of rationality that is instrumental for almost all purposes: this form of rationality is contained in the idea of truth-conduciveness in the explained sense. *This* is the practical reason why it makes sense to *separate* epistemic from non-epistemic goals, and regard the satisfaction of epistemic goals as a good, independent of the practical goals which one actually pursues. I say “for almost all purposes” because there are some important exceptions which I will discuss later. First let me briefly explain—or since this is so obvious, I should better say: recall—why truth-conduciveness is all-purpose instrumental.

Maximizing the utility of one's practical actions (whatever they are) is usually explicated in terms of a *decision situation*. The task is to choose that action among a possible set of competing actions which has the maximum expected utility. Therefore, each decision problem can be reduced to a prediction problem whose task it is to predict which of the possible actions will lead to a maximal expected payoff (in Schurz, 2012 this method is used to reduce action games to prediction games). To predict the expected payoff of the available actions, it is necessary to predict the environmental conditions under which the actions will take place, and the consequences of each action under these conditions. In this way, practical success in a given decision problem depends on cognitive success in a corresponding prediction problem. Therefore, increased success in one's predictions, as measured by the goal of truth, will by and large lead to increased success in one's practical actions, *independently of the goals which one pursues*.

Elqayam and Evans reject truth-conduciveness as the supreme cognitive goal for reasons which do not really conflict with my arguments. They understand the notion of truth in a much more “metaphysical” and less practical and empirical sense than I do. For example, they argue that “cognitive representations are viewed not as veridical, but as fit for purpose” (Elqayam and Evans, 2011, p. 278). This is nothing but the teleological position explained above. They continue with remarking that there is no “true picture of the world which our eyes and brains deliver faithfully to us. There is a mass of information in light, which could be interpreted and constructed in many ways. In addition, our visual systems have clear limitations.” From a philosophical standpoint all of this is obviously true. But this only means that we never know the *complete* truth (“true picture”) of our environment, and that our cognitive models are never free from *simplification* and *error*. However, all that counts for practical success is true information about practically relevant questions, for example whether or not it will rain tomorrow, or whether the value of a given share will go up or down. By “cognitive success” I do *not* mean the achievement of fancy metaphysical truths, but (at least primarily) the achievement of *empirical* (i.e., possibly observable) truths, which are of possible relevance for our practical success. This position is not far from Elqayam and Evans, who infer from their considerations that “cognitive representations are only veridical to the extent and in the manner required to serve our goals.” In conclusion, I am inclined to think that Elqayam and Evans' “instrumental rationality” along with Todd and Gigerenzer's “adaptive rationality” and Evans and Over's “rationality_1_” can be subsumed under the family of a-posteriori conceptions of rationality, which evaluate rationality in terms of their cognitive success in the sense just explained.

Let me finally mention the *big exception* to the all-purpose instrumentality of truthful beliefs. Our beliefs may have certain *direct* effects on us that are quite independent from their truth value. If I believe that a beloved person will visit me in an hour, then this belief makes me happy for the next hour, quite independently of whether or not this person actually comes. Schurz (2001a) calls these effects the *generalized placebo effects* of our beliefs. Placebo effects have been extensively studied in the area of medicine and pharmaceutics. For example, the mere belief in the effectiveness of a sleeping pill accounts for more than 50% of the success of a real sleeping pill. More generally, positive illusions have positive effects on a person's physical and mental health (Taylor, 1989, p. 49, 88ff, 117ff). Particularly effective in this respect are *religious* beliefs. Because of their selective advantages, generalized placebo effects are to some certain extent built into our cognitive processes and are the reason for certain cognitive “biases” that have been discovered in the heuristics-and-biases research in psychology. Piatelli-Palmarini (1996) classifies these cognitive biases in seven groups, where at least three of them are the result of genetically selected placebo effects: overconfidence, hindsight bias, and self-righteous bias.

Placebo effects are *real* and *useful* effects, being produced by one's strong belief in some usually false state of affairs, for example in one's own superiority or in the existence of a safeguarding God. However, placebo effects break down as soon as one comes to believe the truth: the resistance of my body against cancer decreases when my doctor tells me that my chances to survive are small (etc.). In conclusion, placebo effects are the big exception to the all-purpose instrumentality of true beliefs.

Let me emphasize that my comment concerning placebo effects is only intended to direct attention to this problem, but not to offer an adequate treatment (or “solution”) as the latter project would exceed the scope of this paper. Rather than offer a solution, I want to conclude my discussion of this problem with the following remark. The unjustified faith in one's beliefs upon which the placebo effect rests is at the same time practically dangerous: it often leads to a dogmatic belief system which resists revision through the scientific procedures of critical testing, and promotes tendencies to solve conflicts by fiat or violence instead of rational reflection. Despite the beneficial aspects of placebo effects, eliminating vulnerability to placebo effects is a *price* that must paid as a means to acquiring a scientific as opposed to a magical belief system—a price that is worth paying, given the general value of truth beliefs for practical action and the dangers of dogmatic belief.

## General vs. locally adapted rationality: not a normative but a descriptive question

In the preceding sections, I investigated the normative side of rationality. I distinguished two accounts of rationality, a-priori intuition-based vs. a-posteriori success-based. To some extent this distinction reflects Elqayam and Evan's (2011) distinction between prescriptive normativism and descriptive instrumentalism, and Todd and Gigerenzer's (2012) distinction between logical and ecological rationality. Both accounts contain *some* normative elements (in this respect I disagree with Elqayam and Evans), which are, of course, much stronger within intuition-based than within success-based accounts. In the former accounts, the normative elements derive from a mixed bag of a-priori intuitions, while in the latter accounts the only element of intuition concerns the acceptance of cognitive success, i.e., practically relevant truthfulness, as the fundamental cognitive goal, which is justified by its almost-all-purpose instrumentality for practical success.

There is, however, a second distinction, that between *logically general* vs. *locally adaptive* accounts of rationality. Todd and Gigerenzer (2012, p. 15) as well as Elqayam and Evans (2011) equate this distinction with the one between a-priori and a-posteriori accounts: for them logically-general accounts would be normatively justified in an a-priori manner, while locally adapted accounts are a-posteriori justified by their cognitive success in a given kind of environment. In my view, these two distinctions should be treated as two *independent dimensions* of classification, for the following reasons. *Firstly*, logico-general systems of reasoning can also be instrumentally justified, by their a-posteriori success in regard to—not specific but varying—environments and cognitive tasks. *Secondly*, the locally adaptive view of rationality can also be quite explicitly normative: Todd and Gigerenzer (2012) is full of recommendations to use frugal locally adapted heuristics instead of general logical tools. *Thirdly*, a local and special-purpose-related cognitive method, such as Kahnemann and Tversky's availability heuristics, can also be justified by a-priori intuitions: this is the way that such heuristics are justified within Cohen's (1981) “reflective equilibrium” account of cognitive rationality.

While the question of deciding between a-priori intuition-based vs. a-posteriori success-based accounts is a *meta-normative* question, concerning the way normative recommendations can be justified, the question of whether logico-general or locally adapted reasoning methods are more successful is a *descriptive* question, that only can be decided by computational and empirical means. To avoid misunderstanding: this question cannot be decided by finding out which cognitive methods are implicitly used by ordinary people when they reason. This would involve an is-ought fallacy, since we should not expect the actual reasoning of humans to always be cognitively successful or optimal. However, the cognitive success of reasoning methods can be studied by means of logical arguments, by mathematical theorems, and by empirical investigations of their performance in simulated and real-world environments. In the following subsections, I will sketch some typical success-based justifications of cognitive methods, both of logico-general methods that are prominent in philosophy, and of locally adapted reasoning methods that have been promoted by defenders of ecological rationality. I will show, in each case, that a closer inspection of these success-based justifications reveals that the cognitive success of the respective methods is limited to certain situations. The presented justifications of the “competing” cognitive methods do not really contradict each other; only their uncritical generalization as “autocratic paradigms” leads to mutual conflict.

### Instrumental justification of deductive reasoning

The standard justification of (classical) deductive reasoning consists in the provable fact that this kind of reasoning *preserves truth with certainty*: in all possible worlds in which the premises of a deductive argument are true, the conclusion of the argument is also true.

First of all, let me try to remove a misunderstanding which is apparently involved in some arguments that set logical and probabilistic accounts of reasoning in opposition to one another. For example, Elqayam and Evans (2011, p. 278) infer from the fact that Bayesian updating is only possible if the probabilities are non-extreme (different from 1 and 0) that truth-preserving deductive inference is in conflict with Bayesian belief-updating. In this argument they equate the truth of a premise with its having an epistemic probability of 1. However, these two things are entirely different: (1) Obviously, truth is different from having an epistemic probability 1, since something can be true despite the fact that I don't believe it, and vice versa. (2) Further, believing that something is true is not the same as believing it with probability 1, because if I am a fallibilist then I will believe that the proposition I believe could be false, which means that I assign to them a high but not maximal probability. Moreover, knowing that a set of premises entails a certain conclusion can be cognitively useful even if my degree of belief in these premises is non-maximal. It is a straightforward theorem of probability theory that the probability of the conclusion of a valid deductive inference must be at least as high as the probability of the *conjunction* of its premises. Many further theorems of this sort have been proved in the literature, for example, that the uncertainty (i.e., 1 minus the probability) of the conclusion must always be greater than or equal to the sum of the uncertainties of the premises (Suppes, 1966, p. 54). In conclusion, the account of deductive reasoning is *not at all in conflict* with the account of probabilistic reasoning (see also the section Instrumental Justification of Probabilistic Reasoning on this point).

But let us ask: what does the truth-preserving nature of deductive reasoning imply regarding the cognitive success and usefulness of deductive inferences? In order to make cognitive use of a deductive inference, two conditions must be satisfied:

(2) *Conditions for the cognitive usefulness of a deductive inference:* (a) It must be possible for a person with “normal” cognitive abilities to achieve reliable beliefs about the truth of each premise, without (b) that the achievement of this belief relies itself on the person's belief in the truth of the conclusion.

Only if these two conditions are satisfied, can the cognitive process of drawing the deductive inference produce a new belief for the given person, which then is at least as reliable (i.e., probable given the evidence) as the conjunction of its premises. Condition (2a) entails that the premises must be consistent. Condition (2b) is violated, for example, in trivial logical inferences such as “p and q, therefore p,” since all persons with normal cognitive abilities will, in the moment in which they start to believe a conjunction of two beliefs, believe each of its conjuncts. This is not the case in more complicated cases of deductive inference: for example, no cognitively normal person will believe that there are infinitely many prime numbers, from the moment that she begins to understand and believe the axioms of Peano arithmetic. In cases of this sort, deductive proofs produce new cognitive insights and, hence, are cognitively useful.

In the last example, belief in the premises (Peano's axioms of arithmetic) are believed based on mathematical “intuition” or postulate. In empirical applications, knowledge of the premises must be based on empirical evidence. Here we meet a further condition for cognitively usefulness. Nontrivial cognitive inferences usually involve conditionals (implications), which in classical logic are *material* conditionals “p→q,” whose truth-table coincides with “¬p or q.” As a consequence, “p→q” follows deductively from “¬p” and from “q.” I call a material conditional *trivially verified* if the belief in it is justified either by the belief in the negation of its antecedent (¬p) or by the belief into its consequent (q). One can easily see that deductive inference from trivially verified conditionals cannot be cognitively useful:

**Table:** 

(3)		*Trivial verification of 1st premise by*:
(a)	Modus p→q	¬p: Then verification of 2nd premise is impossible
	Ponens: p	q: Then conclusion is already known: inference trivial
	q	
(b)	Modus p→q	q: Then verification of 2nd premise is impossible
	Tollens: ¬q	¬p: Then conclusion is already known: inference trivial
	¬p	

Similar considerations apply to more complicated inferences (see footnote 10), for example to inferences from disjunctions, such as disjunctive syllogism: “p∨q, ¬p, therefore q.” It follows that deductive inferences can only be cognitively useful if their conditional or disjunctive premises are not known by trivial verification. In empirical (non-mathematical) domains the standard way of justifying a *singular* material conditional without knowing the truth value of its if-part and then-part is to infer it from a corresponding *general* conditional, which is in turn *inductively* inferred from the empirical evidence. For example, when I believe that “if Jonny promises to come, then he will come,” I don't believe this because Jonny didn't promise to come or because he actually came, but because I inferred this prediction from his promise-keeping behavior in the past.

In classical logic one can only express *strictly* general conditionals, which don't admit of exceptions and have the form “For all x: Fx→Gx” (with “Fx/Gx” for “x has property F/G”). With their help, the inference in (3a) is transformed into the following:

**Table:** 

(4) *Modus Ponens from the instantiation of a strictly universal conditional*:	
For all x: Fx→Gx	Nontrivial confirmation of the 1st premise by a sample of Fs all of
a is an F	which are Gs, where this sample doesn't contain individual a.
a is a G	

It follows from these considerations that in application to empirical knowledge, the successful cognitive use of deductive inferences is usually ^9^ restricted to *situations* which satisfy the following two conditions:

- The inference contains at least one conditional (or disjunctive) premise which is explicitly or implicitly ^10^ general and can only be confirmed by an inductive inference, and
- the generality of this premise is strict (i.e., exceptionless).

Condition (A) implies that without the simultaneous capacity to reason inductively, deductive inferences are of almost no use in empirical domains. So condition (A) alone is sufficient to refute the view that deductive logic is an all “all-purpose” system of reasoning: although its inferences are truth-preserving in all possible situations (or worlds), they are not cognitively useful in all possible situations^11^, but only in those situations in which deductive reasoning competence is *paired* with success in inductive reasoning.

I assume that for the majority of readers nothing of what I have said is substantially new. But if this is so, I cannot understand how one can seriously regard deductive logic as *the only* normative standard of reasoning. I guess that many of the hegemony claims made on the behalf of given “normative systems” are more the result of power struggles between Kuhnian “paradigms” than of rational reflection.

Condition (B) is an equally severe restriction on the cognitive use of deductive logic. Apart from laws in classical physics, there are not many true and strictly general laws in the empirical sciences. Most empirical conditionals are *uncertain* and admit of exceptions; they have the form “Most Fs are Gs,” or “Normally, Fs are Gs” (cf. Schurz, 2001b, 2002). Conditionals of this sort are usually reconstructed as expressing high conditional probabilities. Reasoning with them requires probabilistic systems, either in the form of a conditional logic based on a probabilistic semantics (cf. Adams, 1975; Schurz, 2005; Schurz and Thorn, 2012; Thorn and Schurz, 2014), or within the full system of mathematical probability theory (see the section Instrumental Justification of Probabilistic Reasoning). Experimental investigations of reasoning have confirmed that people frequently understand uncertain conditionals in the sense of high conditional probabilities (Evans et al., 2003; Schurz, 2007). We will see below, however, that the application of probability theory (or of more advanced mathematical theories) to empirical domains is also only cognitively successful if it is paired with inductive reasoning mechanisms which can provide empirical confirmation for the general premises.

Note that conditions (A) and (B), above, are less restrictive than it may seem. First of all, conditions (A) and (B) do not apply to mathematical domains, where strictly general premises are given by axiomatic stipulation. No wonder, therefore, that deductive inferences are most intensively used in the mathematical sciences. Secondly, the fact that inferences from uncertain scientific laws require the use of probability theory does *not* make deductive logic disappear, because probability theory is usually formalized within standard type-free (Zermelo Fraenkel) set theory, which contains in its core the full power of deductive logic, which is needed, for example, to prove probability theorems from probability axioms. Replacing mere logic by advanced mathematics only breaks the autocracy of logic, but not its omnipresence: all higher-level mathematical theories still contain logic in their core. In conclusion, standing on its own legs deductive logic is highly useful in mathematical domains. In empirical applications, however, its cognitive use is confined to situations in which deductive reasoning is combined with the results of inductive reasoning procedures.

### Instrumental justification of probabilistic reasoning

The instrumental justification of probabilistic reasoning in terms of cognitive success depends on the assumed conception of probability: *statistical* (objective) or *epistemic* (Bayesian, subjective)^12^. The *statistical* probability of a property or event-*type* Fx, p(Fx), is the *limit of its relative frequency* in an underlying *random sequence* consisting of the consecutive outcomes of a random experiment (important founders are Von Mises, 1964, and Fisher, 1956). On the other hand, the *epistemic* probability of a particular state of affairs or event-*token* Fa, P(Fa), is the degree of belief, to which a given rational subject, or all subjects of a certain rationality type, believe in the occurrence of the event (important founders are Bayes, 1763; Ramsey, 1926; De Finetti, 1937).

The standard justification of Bayesian (i.e., epistemic) probabilities is their interpretation as fair betting quotients. Ramsey and de Finetti proved that a bettor's fair betting quotients satisfy the (standard Kolmogorovian) probability axioms if and only if they are *coherent* in the sense that there is no finite class of fair bets which under all possible circumstances lead to a total loss for the bettor. According to this view, the cognitive usefulness of coherent degrees of beliefs consists in the avoidance of sure loss, independent from the given environment. Although I do not deny that this form of probabilistic consistency is of “some use,” it is certainly not enough for truthful prediction or successful action in the *actual* world. The definition of coherent fair betting quotients refers solely to the *subjective mental state* of the betting persons, but it need not reflect the true frequencies of the bet-on events. Take for example a subjective Bayesian who offers odds of 1:1 that she will roll a six with a normal die, and considers the bet fair, i.e., she is willing to accept the opposite bet at 1:1 that she won't roll a six. The Bayesian remains coherent even after she has lost her entire fortune. She may be puzzled that while everybody has readily accepted her bet, nobody has accepted the counterbet, but she can't explain why she of all people has lost everything while others have made their fortune, *as long as* she doesn't consider the frequentistic chances of the type of event she has been betting on. This shows that Bayesian coherence provides at best a *minimal* condition for rational degrees of belief, which is, however, too weak to exclude irrational betting behavior from an objective point of view.

In other words, subjective degrees of belief can *only* be cognitively successful if they are related to statistical probabilities. The most important connection between subjective and statistical probabilities is expressed by a principle that goes back to Reichenbach (1949) (cf. Schurz, 2013, p. 132):

(5) *Principle of narrowest reference class:* the subjective probability P(Fa) of a single event Fa is determined as the (estimated) *conditional* statistical probability p(Fx|Rx) of the corresponding type of event Fx in the *narrowest* (nomological) reference class Rx, within which we know a lies (i.e., that Ra is true).

The principle of the narrowest class of reference (also called the “statistical principal principle”; Schurz, 2013, p. 262) is widely used both in everyday life and in the sciences. If we want to determine the subjective probability that a certain person will take a certain career path (Fa), then we rely on the characteristics of this person which are known to us as the narrowest reference class (Ra), and on the statistical probability that a person x with the characteristics Rx will take this career path (p(Fx|Rx)). The weather forecast “the probability that it will rain *tomorrow* is 3/4” has, according to Reichenbach's principle, the following interpretation: the statistical probability that it will rain on a day which is preceded by similar weather patterns as that preceding today is 3/4. Unterhuber and Schurz (2013, sec. 4.3) argue that Oaksford and Chater (2007), too, seem to accept a principle of this sort.

Bayesian probabilities can only be truth-conducive and cognitively useful if they are connected with statistical probabilities. Only if we can reliably predict the true success probabilities of our actions can our actions be useful. However, all knowledge about statistical probabilities must be inferred from observations of past instances or samples by means of *inductive* inferences. So our conclusion concerning the cognitive usefulness of probability theory is similar to our conclusion for deductive logic: in application to empirical domains, probabilistic reasoning is only useful if it is combined with the capacity for successful inductive inference. In fact, there exists a manifold of accounts which explicate different forms of inductive inference in probabilistic ways—for example, Fisher's, and Neyman and Pearson's account of statistical tests, Fisher's account of statistical inference based on confidence intervals, the approach of Bayesian statistics based on the updating of prior distributions, etc. (for an overview cf. Schurz, 2013, ch. 4). Although it is not possible to enter into the details here, we note that all of these accounts assume *special* principles or rules that go beyond the basic axioms for coherent probabilities and correspond to different forms of inductive inference.

### Instrumental justification of inductive inference: locally adapted methods

In the two preceding subsections we have seen that classical logic and probability theory are far from being “all-purpose” cognitive tools. To be sure, deductive inferences are truth-preserving and coherent probabilities avoid sure-loss, but beyond that, the two reasoning systems can only be successful in empirical applications if they are paired with successful inductive inferences. It is the very domain of inductive inferences, however, in which no universally reliable method, nor even a universally optimal method, exists. Negative results of this sort basically go back to the insights of the philosopher David Hume and have more recently been proved in the areas of formal learning theory (Kelly, 1996), machine learning (Cesa-Bianchi and Lugosi, 2006), and meta-induction (Schurz, 2008; Vickers, 2010, §6.3). This is not to deny that in the area of inductive prediction there are a variety of positive results, but they either hold only under restrictive conditions, or they hold only in the “infinitely long run,” and tell us nothing about the cognitive success of a respective method in practically relevant time. So very naturally, inductive prediction tasks have been the domain in which the paradigm of *locally adaptive* or *ecological* rationality has emerged, which has been developed, among others, by Gigerenzer, Todd, and the *ABC research group*^13^. These researchers show, based on comparative investigations of the success of different prediction methods, that simple prediction heuristics are frequently more successful than more general and computationally costly prediction mechanisms, following the slogan “less can be more.” Gigerenzer et al. (1999) have studied several different heuristics at different levels of generality. In this subsection I focus my discussion on the performance of one of these prediction rules, known as “take-the-best” (TTB).

The prediction tasks studied within the ABC research group have the following format: prediction methods are based on so-called *cues* C1,…,Cn, which are themselves *predictive indicator*s of a criterion variable X whose values or value-relations have to be predicted. Each cue has a given *probability* of predicting correctly, conditional on its delivering any prediction at all. This conditional probability is called the cue's ecological *validity*. In one of the typical experiments, the task was to predict which of two German cities has a higher population, based on binary cues such as (C1) is it a national or state capital?, (C2) does it have a first division soccer team?, etc. In experiments of this sort, a cue (Ci) delivers a prediction if it “discriminates” between the two compared objects: if the cue difference is +1 (value 1 for city A and 0 for city B), the cue predicts X_A_ > X_B_ (city A is larger than city B); if the cue difference is −1 (value 0 for city A and 1 for B), it predicts X_B_ > X_A_ (city B is larger than A), and otherwise it fails to predict.

For each item (i.e., pair of cities), the strategy TTB predicts what the cue with the highest ecological validity predicts, among all cues which deliver a prediction for the given item. The frugality of this strategy consists in the fact that for each item it bases its prediction on only one cue (that with the highest validity). In contrast, more complex strategies predict a certain (mathematical) combination of the predictions of all cues. For example, the strategy called “Franklin's rule” predicts according to a weighted average of the cue differences of all discriminating cues, where the weights are determined by the (normalized) validities of the discriminating cues (Gigerenzer et al., 1999, part III). If this weighted average is greater (or smaller) than 0.5, Franklin's rule predicts X_A_ > X_B_ (or X_A_ < X_B_, respectively). A still more complex prediction method is linear (or logistic) regression: this method predicts a linear (or logistic) combination of the cue differences with optimal weights which minimize the sum of squared distances between the actual value of the item (which takes +1 if X_A_ > X_B_ and −1 if X_A_ < X_B_) and the predicted linear (or logistic) combination of cues (Gigerenzer et al., 1999; Rieskamp and Dieckmann, 2012).

It can be proved that both regression methods have equally maximal predictive success among all linear combinations, if their weights are fitted to 100% of all items of the underlying population (in our example all pairs of cities). In practice, however, the weights are estimated from so-called *training sets*, which consist of random samples of varying size (e.g., 20% of all items). Likewise, Franklin's rule and TTB estimate the validities of the cues from their validities in training sets. This is the point where the advantage of frugal strategies such as TTB comes in. Regression methods, and to some extent also Franklin's rule, suffer frequently from the problem of *overfitting*: they fit the weights or validities to random accidentalities of the sample which disappear ‘in the long run,’ when the samples size approaches the population size (cf. Brighton and Gigerenzer, 2012, Figures 2–1; Rieskamp and Dieckmann, 2012, p. 198f, Figures 8–1, 8–2). Based on simulated and real data, Rieskamp and Dieckmann (2012) arrive at the result that linear weighting methods tend to be better than TTB in environments of low redundancy, with little (unconditional) correlations between the cues' predictions, while in high redundancy environments TTB tends to be better than weighting methods (for small learning samples) or equally good (for large learning samples)^14^. However, one can show that Rieskamp and Dieckmann's generalizations from their empirical results are not always correct. Schurz and Thorn (in review) construct environments in which weighting rules are superior in spite of redundant cues, as well as environments in which TTB is superior in spite of non-redundant cues (see Appendix). In the next section we will see that there is a systematic reason for the difficulty of providing simple rules that characterize the class of environments in which frugal prediction methods such as TTB beat complex prediction methods such as Franklin's rule or regression.

## A dilemma for ecological rationality, or why a dual account is needed

The success of any locally adapted prediction method depends on its being applied in the “right” *environment*. However, biological organisms, and especially humans, frequently face *changing* environments. Within such environments, one needs strategies that *select* for each relevant environment a method, or a *combination* of methods, that performs as well as possible in that environment. Following Rieskamp and Otto (2006, p. 207), I call this the *strategy selection* problem.

Researchers within the adaptive rationality program acknowledge the importance of the strategy selection problem. For Todd and Gigerenzer (2012 p. 15), the study of ecological rationality centers around the question of which heuristics are successful in which kinds of environments. They propose a list of simple rules which indicate, for each of their studied heuristics in which kind of environment it may be successfully applied, and in which it may not (Todd and Gigerenzer, 2012, Table 1.1). On closer inspection, however, their rules are problematic, either because their application requires information that is unlikely to be available, or because the rules are not always correct. For example, the recognition heuristic (“base your prediction on that cue which is best recognized by you”) is said to be ecologically rational if its ecological validity is greater than 0.5. But since this ecological validity is unknown in advance and only learnable in retrospect, this kind of selection rule is not very helpful. Moreover, the rule is incorrect inasmuch as anybody who possesses a better method than the recognition heuristic should apply this method instead of the recognition heuristic. Concerning the take-the-best heuristic TTB, Todd and Gigerenzer (2012, p. 9) assert (like Rieskamp and Dieckmann, 2012) that TTB is ecologically rational in environments with high cue redundancy and highly varied cue validities, while linear weighting-rules are said to be rational in the opposite types of environments. However, as explained in the preceding section, the connection between high cue redundancy and TTB's optimality can be violated in both directions (see Appendix); so this rule is also incorrect.

The preceding observations do not diminish the great success of the adaptive rationality program in discovering surprising “less is more” effects. They rather point toward an underdeveloped area in this program, namely the selection-of-methods problem. They also indicate a major challenge, and to a certain degree even a *dilemma*, for the program of ecological rationality. For *if there were* simple rules of the form “In environment of type E_i_, method M_i_ is optimal” (for i ∈ {1,…,n}), then the combined strategy “For all i∈{1,…,n}: apply method M_i_ in environment E_i_” would be a universally optimal strategy. The existence of such a strategy would, thereby, *re-install* universal rationality, and *undermine* the very program of adaptive rationality.

Can universal rationality be re-installed in this simple way? The answer is: *No*. Following from well-known results in formal learning theory (Kelly, 1996) and meta-induction (Schurz, 2008), there cannot be an inductive prediction or inference method which is optimal in *all* environments among *all* possible prediction methods. This fact has been frequently mentioned by researchers within the adaptive rationality program (cf. Todd and Gigerenzer, 2012, p. 5). A consequence of the cited result is that there cannot be exhaustive and fully general meta-rules which specify for each task and environment a locally optimal method. Schurz and Thorn (in review) call this fact the *revenge of ecological rationality*.

While there is no ‘absolutely’ optimal selection strategy, the ecological rationality program presupposes selection rules that are at least “very” or “sufficiently” general. Obviously, selection strategies can only have a cognitive benefit if their success is highly general, applying to a large class of environments and tasks. If such general selection strategies did not exist, one could not explain why humans are so successful in selecting the ‘right’ method for their given environment, in spite of the fact that their environment constantly changes.

What makes it difficult to find general rules for selecting methods is that the *success-relevant features* of the environment are frequently cognitively inaccessible. Similarly, *changes* in the environment are often unrecognizable and unforeseeable. To deal with changing environments of this sort, one needs strategies for *learning* which locally adapted methods perform best in which environment, or in which temporal phases of the environment. This brings us to the account of strategy selection by *learning* proposed by Rieskamp and Otto (2006) and the more general account of *meta-induction* developed in Schurz (2008) and Schurz and Thorn (in review). While Rieskamp and Otto suggest *reinforcement* as the learning method for strategy selection, *meta-induction* is a more general family of meta-level selection strategies which includes reinforcement as a special case.

The account of meta-induction was developed within the domain of epistemology as a means of addressing Hume's problem of induction (Schurz, 2008, 2009; Vickers, 2010), thereby utilizing certain results from the domain of machine learning (Cesa-Bianchi and Lugosi, 2006). In this account, meta-inductive selection strategies are considered as *meta-level* strategies. Such strategies attempt to select an optimal prediction method, or to construct an optimal *combination* of such methods, out of the *toolbox* of locally adapted prediction methods, which are also called the object-level methods. Meta-inductive strategies base their predictions on the *so-far observed success rates* of the available object-level methods. The simplest meta-inductive strategy is again TTB, which imitates the predictions of the so-far best available prediction method. The difference between the model envisioned here and the typical experimental paradigm used within adaptive rationality research is that within the present model TTB is applied at the meta-level, as a means to selecting the right (combination of) locally adapted prediction methods, rather than to the selection of “cues.”

Recall the negative result that there is no method which is optimal among *all* possible prediction methods in all environments. In other words, no method is *absolutely* optimal. Of course, only a fraction of all possible prediction methods is cognitively accessible to any human-like agent. So at the meta-level, it is only possible to include *the cognitively accessible* prediction methods in the “toolbox” of candidate methods. This raises the following question: is there a meta-inductive strategy which predicts optimally in comparison to all candidate prediction methods that are *accessible* to it, no matter what these methods are and in which environment one happens to be? Schurz and Thorn (in review) call this property *access-optimality* (i.e., optimality among all accessible methods), in distinction to absolute optimality, which is not restricted to the accessible methods.

The philosophical importance of this notion is this: if one could prove that a universally access-optimal selection strategy exists, its application would always be reasonable, independent from one's environment and one's toolbox, because by applying this meta-strategy to the methods in one's toolbox, one can only improve but never worsen one's success rate. Arguably, the existence of such a method would also give us at least a partial solution to Hume's problem of induction (Schurz, 2008). It can easily be shown that TTB is *not* universally access-optimal: it fails to be access-optimal in environments where the success rates of the available candidate methods are constantly oscillating (Schurz, 2008, Figures 1, 4). However, there is a certain linear weighting strategy, so far unrecognized within the adaptive rationality research community, which is demonstrably universally access-optimal *in the long run*. Schurz and Thorn (in review) call this strategy *attractivity-based* weighting, AW, since it bases its assignment of weights to the predictions of accessible methods on the “attractivities” of those methods, which depend on the success differences between the object-level methods and AW. In the *short* run, AW may earn a small loss (compared to the so-far best prediction method) which vanishes if the number of rounds becomes large compared to the number of competing methods.

There are meta-level methods whose performance exceeds that of AW in particular environments. For example, Schurz and Thorn (in review, sec. 7) show that Franklin's rule (if applied at the meta-level) outperforms AW in certain environments, but is worse than AW in other environments. In other words, all improvements of AW are *local* and come at the cost of losing universal access-optimality. This is again a “revenge effect” of ecological rationality, which puts us into the following dilemma: on the one hand, there is a meta-level strategy, namely AW, which is universally access-optimal in the long run. On the other hand, there are methods whose performance may exceed that of AW locally, but only on the cost of losing universal access-optimality.

Schurz and Thorn (in review) propose to solve this dilemma by the following *division of labor*: At the meta-level of selection strategies, one should use a strategy which is access-optimal, i.e., the strategy AW. If one finds a meta-method M^*^ which is more successful than AW in some environments, then one can improve the success of AW not by replacing AW by M^*^ at the meta-level, but by putting M^*^ into the toolbox of locally adapted methods and applying AW to this *extended* toolbox.

Generally speaking, the account of Schurz and Thorn (in review) proposes a division of labor between *general* meta-level selection strategies and optimal (combinations of) *locally adapted* cognitive methods. This proposed division of labor is akin to the *dual process accounts of cognition* that have been developed in the recent decades by a variety of psychologists^15^. These accounts explain human cognition by a division of labor between two reasoning systems and corresponding processes: “type 1” processes are usually characterized as unconscious or implicit, heuristic, context-specific, perception- or action-related, fast and parallel, and evolutionarily old (humans share them with animals). In contrast, “type 2” processes are characterized as conscious and explicit, analytic, context-general, symbolic, slow and sequential, being an evolutionarily recent feature of homo sapiens.

Although the fit of the dualistic account of local methods and meta-inductive strategies with dual process accounts is not perfect, the basic similarities are clear. *Firstly*, the distinction between locally adapted prediction strategies and general (meta-inductive) selection strategies is a distinction between types of cognitive *processes* (not between cognitive “rationalities”); so it rightly belongs to the cognitive process level to which the type 1/2 distinction applies (cf. Oaksford and Chater, 2012). *Secondly*, meta-inductive strategies are conscious selection processes and thus belong to the family of type 2 processes. In contrast, locally adapted prediction or decision heuristics are often (though not always) type 1 processes, whose control by type 2 processes is difficult and requires cognitive training (Houde et al., 2000) and general intelligence (Stanovich, 1999).

The preceding short remark concerning the relation between the proposed dual account and contemporary dual process theories must be sufficient. The main purpose of the dual account of local methods and meta-strategies is to highlight the evolutionary benefit of a division of labor between general cognitive selection strategies and locally adapted cognitive methods. In particular, this division of labor helps to solve the explained dilemma facing ecological rationality program, i.e., the problem of explaining how locally adapted reasoning strategies can be cognitively successful in a situation of changing environments.

## Conclusion

I began this article by outlining the distinction between normative and instrumental conceptions of cognitive rationality (Elqayam and Evans, 2011). The latter distinction was embedded into a broader philosophical framework, classifying the former account as deontological and the latter as teleological. While I agreed with Elqayam and Evans' critique of unjustified is-ought inferences in normative accounts, I argued that in both accounts one must make at least some value assumptions, which are based on some form of intuition. However, while those accounts which Elqayam and Evans call “normativist” are based on a mixed bag of a-priori intuitions, instrumentalist accounts are based on just one value—the value of cognitive success in the given environment. I, therefore, proposed to replace the normative/instrumental distinction by the distinction between a-priori intuition-based vs. a-posteriori success-based accounts of rationality. Cognitive success should be understood as success in finding as many relevant truths as possible with as few mistakes and cognitive costs. I argued that the value of cognitive success lies in its instrumental rationality for *almost-all* practical purposes.

After distinguishing between a-priori intuition-based vs. a-posteriori success-based accounts of rationality, I pointed out that this distinction is usually conflated with a second distinction: that between logically general vs. locally adapted rationality. In opposition to this conflation, I argued that these two distinctions should be treated as *independent* dimensions of classification. The question of whether logico-general or locally adapted reasoning methods have greater cognitive success is a descriptive question which can be decided by computational and empirical means. In the case of classical logic and probability theory, I demonstrated that logico-general systems of reasoning can be instrumentally justified by their a-posteriori cognitive success. It turns out that although reasoning according to classical logic and probability theory have the advantage of preserving truth and avoiding inconsistency in all environments, they are not cognitively successful in all situations, but only in those where they are paired with capacity for successful inductive reasoning, which supplies deductive or probabilistic reasoning with general premises about empirical regularities. In the area of inductive inference, however, there is no generally reliable or optimal reasoning method. No wonder, then, that this is the domain in which the paradigm of locally adaptive or ecological rationality has emerged.

In the final part of the paper, I argued that the fact that human beings frequently encounter changing environments generates a dilemma for the program of ecological rationality. On the one hand, this program requires rules which specify for each heuristic the kind of environment in which it may be successfully applied, and in which it may not. On the other hand, some general arguments show that a complete list of rules of this sort does not exist; in fact, its existence would undermine the very program of ecological rationality. As a way out of this dilemma, I argued for a dual account of cognition, in which highly general meta-inductive selection strategies are applied to a toolbox of locally adapted cognitive methods.

### Conflict of interest statement

The author declares that the research was conducted in the absence of any commercial or financial relationships that could be construed as a potential conflict of interest.

## Figures and Tables

**Figure A1 + A2:**
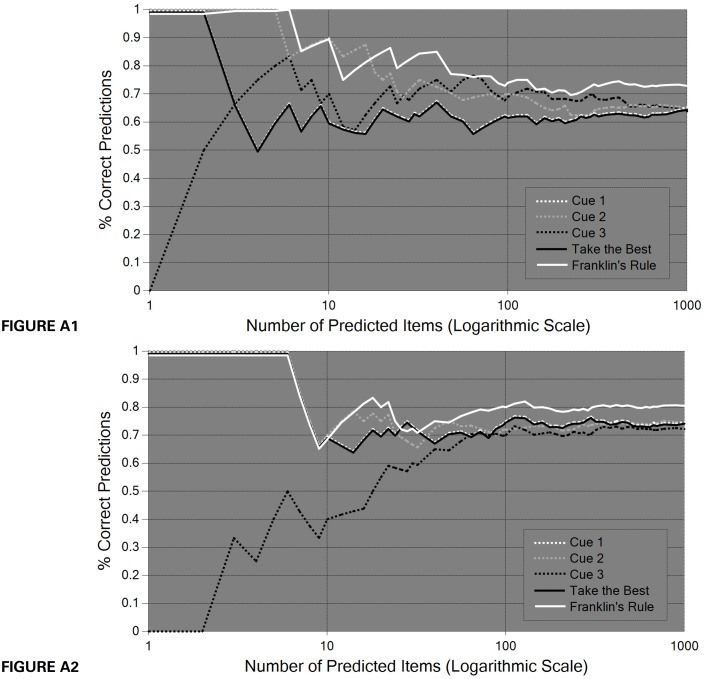
**TTB against Franklin's rule in a binary prediction tournament with known validities which are conditionally dependent**. Figure A1 with low redundancy and Figure A2 with high redundancy of cues. In both cases, Franklin's rule predicts better than TTB.

**Figure A3 + A4:**
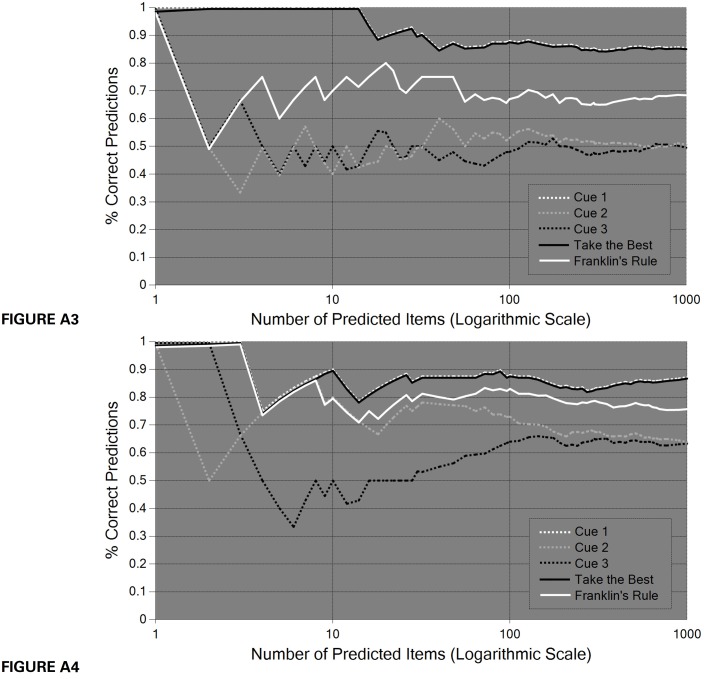
**TTB against Franklin's rule in a binary prediction tournament with known validities which are conditionally independent**. Figure A3 with low redundancy and Figure A4 with high redundancy of cues. In both cases TTB predicts better than Franklin's rule.

## Footnotes

^1^A forerunner is Evans and Over's notion of “rationality_2_” (1996, p. 8).

^2^Elqayam and Evans (2011), p. 237f; in particular p. 277 in reply to Schurz (2011a).

^3^Cf. Gigerenzer et al. (1999), Todd and Gigerenzer (2012), and Evans and Over (1996).

^4^Cf. Schurz (1997): for (H1) theorems 3–5 (p. 118, 121, 124), for (H2) theorems 1–2 (p. 92, 102), for (H3) prop. 7 (p. 74). For related work cf. Stuhlmann-Laeisz (1983), Pigden (1989), Galvan (1988); general introductions are Hudson (1969) and Pigden (2010).

^5^For example, for the purpose of letting fresh air into the room, tearing down a wall is a sufficient but neither a necessary nor an optimal means. Some people object that the means-end inference fails even for necessary (or optimal) means, since if the necessary means B for realizing the fundamental norm A is itself intrinsically bad, B should not be realized. However, in such a case it is unreasonable to accept A as a fundamental norm. Thus, although this objection points to an important constraint on fundamental norms, it fails as an argument against the means-end inference.

^6^Cf. Moore's famous “open question” argument against the “naturalistic fallacy” of defining “Ought” by “Is” (1903, p. 15f).

^7^Goldman (1986) is an exception among epistemologists inasmuch as he also discusses cognitive costs.

^8^This bridge principle says: “If person X has goal A, and A is not in conflict with other goals of X, then A's realization is valuable for X” (cf. Schurz, 1997, sec. 11.7).

^9^A generalization to deductive inferences of arbitrary kind is possible by the following consideration: Statements which are verifiable by observation have the form of closed literals, i.e., unnegated or negated statements of the form (¬)Fa, or (¬)Rab, etc. (where F, R, etc. are primitive non-logical predicates). However, deductive inferences among literals necessarily fail to meet condition (2b) of cognitive usefulness, because a literal follows from a set Δ of literals if and only if it is an element of Δ [cf. (Schurz, 2011b), sec. 5.1, (5)].

^10^The conditional premise Fa→Ga is said to be *implicitly general* if it is justified by an argument which justifies a corresponding conditional Fa_*i*_→Ga_*i*_ *for every other individual constant* a_*i*_. For example, Fa→Ga is implicitly general if it is deductively inferred from the explicitly general premise ∀x(Fx→Gx), or if it is inductively inferred from sample information of the form {Fb_1_→Gb_1_, …, Fb_n_→Gb_n_}.

^11^My notion of a *situation* includes both (a) an objective (subject-independent) environment, and (b) a constellation of subjective facts concerning the given cognitive task and cognitive resources.

^12^We confine our discussion to these two most important conceptions of probability. Further probability concepts which we cannot discuss here are objective single case probabilities and logical probabilities. Cf. Gillies (2000, ch. 3.13).

^13^Cf. Gigerenzer et al. (1999) and Todd and Gigerenzer (2012). “ABC” stands short for the “Center for Adaptive Behavior and Cognition” at the MPI for Human Development in Berlin.

^14^Moreover, Rieskamp and Dieckmann report that in environments of low redundancy, TTB performs better if the dispersion of the cues' validities is high. Logistic regression performs better than Franklin's rule for training set sizes of greater than 20%, except in environments of high redundancy and low validity dispersion, in which logistic regression beats the other methods only for training sets greater than 80%.

^15^Cf. Evans and Over (1996), Sloman (1996), and Stanovich (1999); for excellent overviews cf. Evans (2003, 2008).

## Appendix: TTB vs. Franklin's rule in environments of different redundancy (with P. Thorn)

Figures A1 + A2 illustrate a simulation of a prediction tournament in which Franklin's rule predicts better than TTB, independently of the cue redundancy of the environment. The value of a binary target variable (with values 1, 0) had to be predicted based on three binary cues (with values 1, 0) whose conditional success probabilities were as follows:

$$\begin{array}{l} p(\text{event}=1|3\text{-of-}3\text{ cues predict }1)=0.9\\ p(\text{event}=1|2\text{-of-}3\text{ cues predict }1)=0.7\\ p(\text{event}=1|1\text{-of-}3\text{ cues predict }1)=0.3\\ p(\text{event}=1|0\text{-of-}3\text{ cues predict }1)=0.1. \end{array}$$

The validities were assumed to be known so that learning errors play no role. By assuming different prior probabilities over the cues' combined predictions, one can make them uncorrelated (non-redundant) or highly correlated (redundant), without changing the result that in this environment Franklin's rule performs better than TTB, as shown in Figures A1 + A2.

Figures A3 + A4 exhibit an environment in which TTB predicts better than Franklin's rule, independently of the degree of cue redundancy. Here the success probabilities of the three cues were as follows (with “C_2/3_” for “cue 2” or “cue 3”):

$$\begin{array}{l} p(\text{event}=1|{\text{C}}_{1}\text{ predicts }1,{\text{C}}_{2/3}\text{ predicts }{\text{x}}_{2/3}\in \{0,1\})\\ \;=0.9\text{ for all choices}]\text{ of }{\text{x}}_{2/3}.\\ p(\text{event}=0|{\text{C}}_{1}\text{ predicts }0,{\text{ C}}_{2/3}\text{ predicts }{\text{x}}_{2/3}\in \{0,1\})\\ \;=0.8\text{ for all choices of }{\text{x}}_{2/3}. \end{array}$$

By assuming either uniform prior distributions over the combined predictions or positive correlations between the cues' predictions one now obtains the result that TTB predicts better than Franklin's rule, both in low and high redundancy environments, as shown in Figures A3 + A4.
